# PDZRN3 protects against apoptosis in myoblasts by maintaining cyclin A2 expression

**DOI:** 10.1038/s41598-020-58116-1

**Published:** 2020-01-24

**Authors:** Takeshi Honda, Makoto Inui

**Affiliations:** 1 Department of Pharmacology, Yamaguchi University Graduate School of Medicine, Ube, Yamaguchi 755-8505 Japan; 2 Present Address: YIC Rehabilitation College, 4-11-1 Nishiube-Minami, Ube, Yamaguchi 759-0208 Japan

**Keywords:** Apoptosis, Cell division

## Abstract

PDZRN3 is a PDZ domain-containing RING-finger family protein that functions in various developmental processes. We previously showed that expression of PDZRN3 is induced together with that of MyoD during the early phase of skeletal muscle regeneration *in vivo*. We here show that PDZRN3 suppresses apoptosis and promotes proliferation in myoblasts in a manner dependent on cyclin A2. Depletion of PDZRN3 in mouse C2C12 myoblasts by RNA interference reduced the proportion of Ki-67-positive cells and the level of Akt phosphorylation, implicating PDZRN3 in regulation of both cell proliferation and apoptosis. Exposure of C2C12 cells as well as of C3H10T1/2 mesenchymal stem cells and NIH-3T3 fibroblasts to various inducers of apoptosis including serum deprivation resulted in a greater increase in the amount of cleaved caspase-3 in PDZRN3-depleted cells than in control cells. The abundance of cyclin A2 was reduced in PDZRN3-depleted C2C12 myoblasts, as was that of Mre11, which contributes to the repair of DNA damage. Overexpression of cyclin A2 restored the expression of Mre11 and Ki-67 as well as attenuated caspase-3 cleavage in PDZRN3-depleted cells deprived of serum. These results indicate that PDZRN3 suppresses apoptosis and promotes proliferation in myoblasts and other cell types by maintaining cyclin A2 expression.

## Introduction

PDZ domain-containing RING-finger protein 3 (PDZRN3 or LNX3) was originally identified *in silico* as a homolog of LNX1 (PDZRN2), whose function as E3 ubiquitin ligase for NUMB is thought to contribute to cell fate determination at the level of inhibition of Notch signaling^1^. Although both LNX1 and PDZRN3 contain an NH_2_-terminal RING domain and a COOH-terminal consensus binding motif for the PDZ domain, LNX1 possesses four PDZ domains in its central region whereas PDZRN3 has only two. We first cloned PDZRN3 cDNA from human heart, and we showed that the protein is also expressed in various other human tissues including skeletal muscle, brain, and liver^2^. Subsequent studies implicated PDZRN3 in developmental processes such as the differentiation of myoblasts^2^, osteoblasts^3^, and adipocytes^4^, neurogenesis^5^, vascular morphogenesis^6^, maintenance of endothelial intercellular junctions^7^, and control both of paracellular Mg^2+^ flux in renal tubular epithelial cells^8^ and of the spatial arrangement of neurons^9^. Mutations of the PDZRN3 gene have also been identified in several tumor types^10–15^.

Regeneration of skeletal muscle is initiated by the activation of satellite cells (myogenic stem cells) in response to injury. These cells give rise to myoblasts that express the transcription factor MyoD, proliferate (early phase of differentiation), and subsequently differentiate into myocytes that express the transcription factor myogenin and fuse to form myotubes (late phase of differentiation). We previously showed that depletion of PDZRN3 inhibited the late phase of differentiation of myocytes into myotubes^2,16^, and that the expression of PDZRN3 is induced together with that of MyoD during regeneration of injured skeletal muscle *in vivo*^2^. The latter finding suggests that PDZRN3 plays a role during the early phase of muscle regeneration in addition to the late phase. However, the function of PDZRN3 in myoblasts has remained unknown. We have now examined the effects of PDZRN3 depletion on the survival and proliferation of C2C12 myoblasts. We found that PDZRN3 regulates apoptosis and proliferation through cyclin A2 in these cells, and that such regulation appears common to other cell types that express PDZRN3.

## Results

### Depletion of PDZRN3 reduces the viability of C2C12 myoblasts

**Figure 1:**
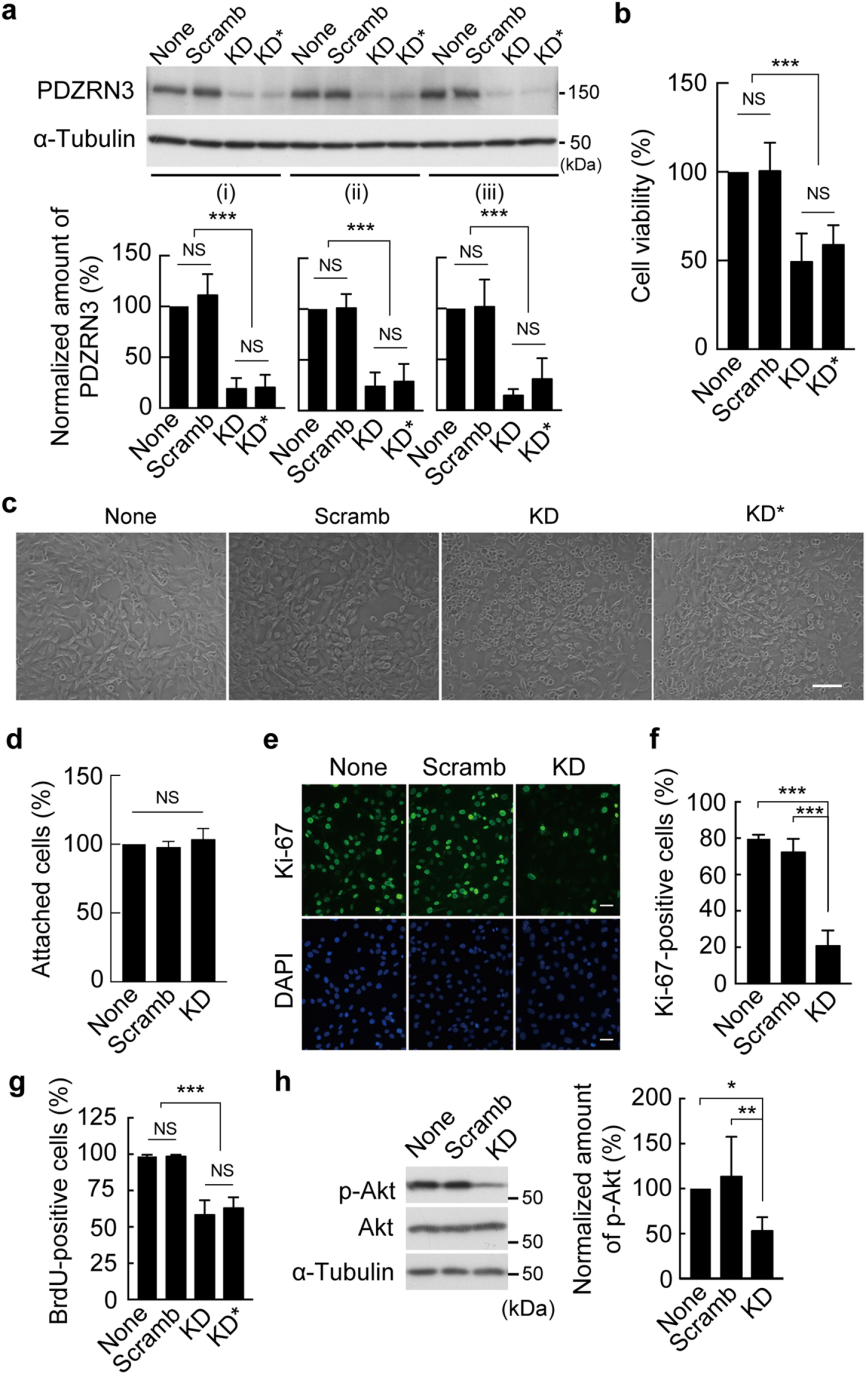
PDZRN3 depletion attenuates proliferation and promotes apoptosis in C2C12 myoblasts. (**a**) Abundance of PDZRN3 in cells infected or not (None) with adenoviruses encoding either a PDZRN3 shRNA (KD or KD*) or a scrambled version of KD (Scramb) and then cultured for 48 h in GM until confluent (i). The cells were then cultured for 24 h in serum-deficient medium (ii) or reseeded and cultured for 48 h in GM until 30% to 40% confluent (iii). Cell lysates were subjected to immunoblot analysis with antibodies to PDZRN3 and to α-tubulin (loading control). A representative blot as well as quantitative data (means ± s.e.m. for five biological replicates) for the abundance of PDZRN3 normalized by that of α-tubulin and expressed relative to the value for noninfected cells are shown. (**b**) Relative viability of cells infected as in (**a**) and then cultured for 48 h was determined by trypan blue staining. Data are means ± s.e.m. for 10 biological replicates. (**c**) Phase-contrast microscopy of cells infected as in (**a**) and then cultured for 48 h. *Scale bar*, 100 μm. (**d**) Relative adhesion activity of cells infected with adenoviruses for KD or Scramb shRNAs was measured 1 h after reseeding. Data are means ± s.e.m. for 10 biological replicates. (**e**) Immunofluorescence analysis of Ki-67 in C2C12 cells infected with adenoviruses encoding KD or Scramb shRNAs. Nuclei were stained with DAPI. *Scale bars*, 25 μm. (**f**) Proportion of Ki-67-positive cells among total (DAPI-positive) cells as determined for images similar to those in (**e**). Data are means ± s.e.m. for five biological replicates. (**g**) The proportion of BrdU-positive cells among total (DAPI-positive) cells infected as in (**a**) was determined by immunofluorescence analysis. Data are means ± s.e.m. for five biological replicates. (**h**) Immunoblot analysis of total and Ser^473^-phosphorylated (p-) forms of Akt in cells infected with adenoviruses for KD or Scramb shRNAs. A representative blot as well as quantitative data (means ± s.e.m. for seven biological replicates) for the relative p-Akt/Akt densitometric ratio are shown. For all panels: *P < 0.05, **P < 0.01, ***P < 0.001; NS, not significant (one-way ANOVA).

We found that RNA interference (RNAi)-mediated depletion of PDZRN3 by infection with an adenovirus encoding a short hairpin RNA (shRNA) specific for PDZRN3 mRNA (KD) in C2C12 myoblasts maintained in growth medium (GM) resulted in a significant decline in cell number compared with control cells infected with an adenovirus encoding a scrambled version of the PDZRN3 shRNA sequence (Scramb) (Fig. 1a,b). Similar results were obtained with a second shRNA specific for PDZRN3 mRNA (KD*). Rounded and detached myoblasts were also more frequently observed in cultures of PDZRN3-depleted cells than in those of control cells (Fig. 1c). Adhesion activity did not differ between PDZRN3-depleted and control C2C12 myoblasts (Fig. 1d). Immunofluorescence analysis revealed that the proportion of Ki-67-positive cells was reduced among PDZRN3-depleted cells compared with control cells (Fig. 1e,f). The incorporation of 5-bromo-2′-deoxyuridine (BrdU) was also reduced in PDZRN3-depleted cells compared with control cells (Fig. 1g). These results thus indicated that depletion of PDZRN3 inhibits cell proliferation. Immunoblot analysis also showed that the extent of Akt phosphorylation at Ser^473^ was significantly reduced by PDZRN3 depletion (Fig. 1h), suggesting that apoptosis is induced as a result of the loss of PDZRN3.

### Depletion of PDZRN3 promotes apoptosis

**Figure 2:**
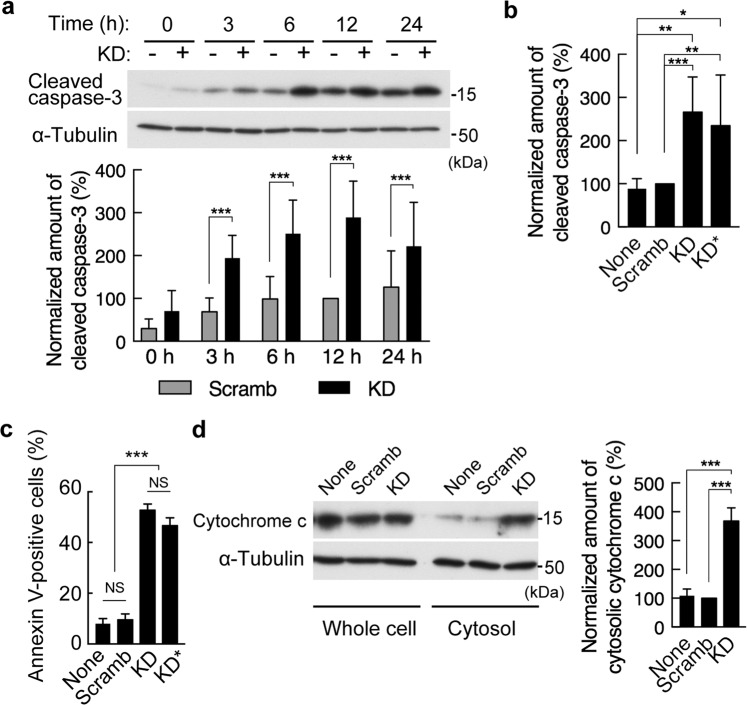
PDZRN3 depletion promotes apoptosis induced by serum deprivation in C2C12 myoblasts. (**a**) Immunoblot analysis of cleaved caspase-3 and α-tubulin (loading control) in C2C12 cells infected with adenoviruses encoding an shRNA specific for mouse PDZRN3 mRNA (KD) or a scrambled version of this shRNA sequence (Scramb) and then subjected to serum deprivation for the indicated times. A representative blot as well as quantitative data (means ± s.e.m. for 10 biological replicates) for the abundance of cleaved caspase-3 normalized by that of α-tubulin and expressed relative to the value for Scramb at 12 h are shown. ***P < 0.001 (two-way ANOVA). (**b**) The experiment in (**a**) was repeated with a different shRNA specific for PDZRN3 mRNA (KD*). Data for cells at 6 h after the onset of serum deprivation are presented as means ± s.e.m. for five biological replicates. *P < 0.05, **P < 0.01, ***P < 0.001 (one-way ANOVA). (**c**) The proportion of early apoptosis (annexin V-positive, propidium iodide-negative) C2C12 cells after infection as in (**b**) and serum deprivation for 6 h. The total cell number was determined by DAPI-staining. Data are means ± s.e.m. for four biological replicates. ***P < 0.001, NS (one-way ANOVA). (**d**) Immunoblot analysis of cytochrome c in whole lysates and a cytosolic fraction of C2C12 cells infected as in (**a**) and deprived of serum for 12 h. Data for the abundance of cytosolic cytochrome c normalized by that of total cytochrome c and expressed relative to the value for Scramb are means ± s.e.m. for five biological replicates. ***P < 0.001 (one-way ANOVA).

To examine further the role of PDZRN3 in apoptotic regulation, we exposed C2C12 myoblasts to serum-depleted medium in order to induce apoptosis. Immunoblot analysis revealed that the amount of cleaved caspase-3, a marker of apoptosis, was significantly higher in PDZRN3-depleted cells than in control cells after serum deprivation (Fig. 2a,b). Immunofluorescence analysis of annexin V, another marker of apoptosis revealed that the proportion of annexin V-positive cells was also larger among PDZRN3-depleted cells compared with control cells after serum deprivation (Fig. 2c). The release of cytochrome c from mitochondria into the cytosol is a key step in the mitochondrial (intrinsic) pathway of apoptosis. We found that the extent of cytochrome c release into the cytosol was also greater for PDZRN3-depleted cells than for control cells subjected to serum deprivation (Fig. 2d). Together, these results thus suggested that PDZRN3 restrains apoptosis in C2C12 myoblasts.

**Figure 3:**
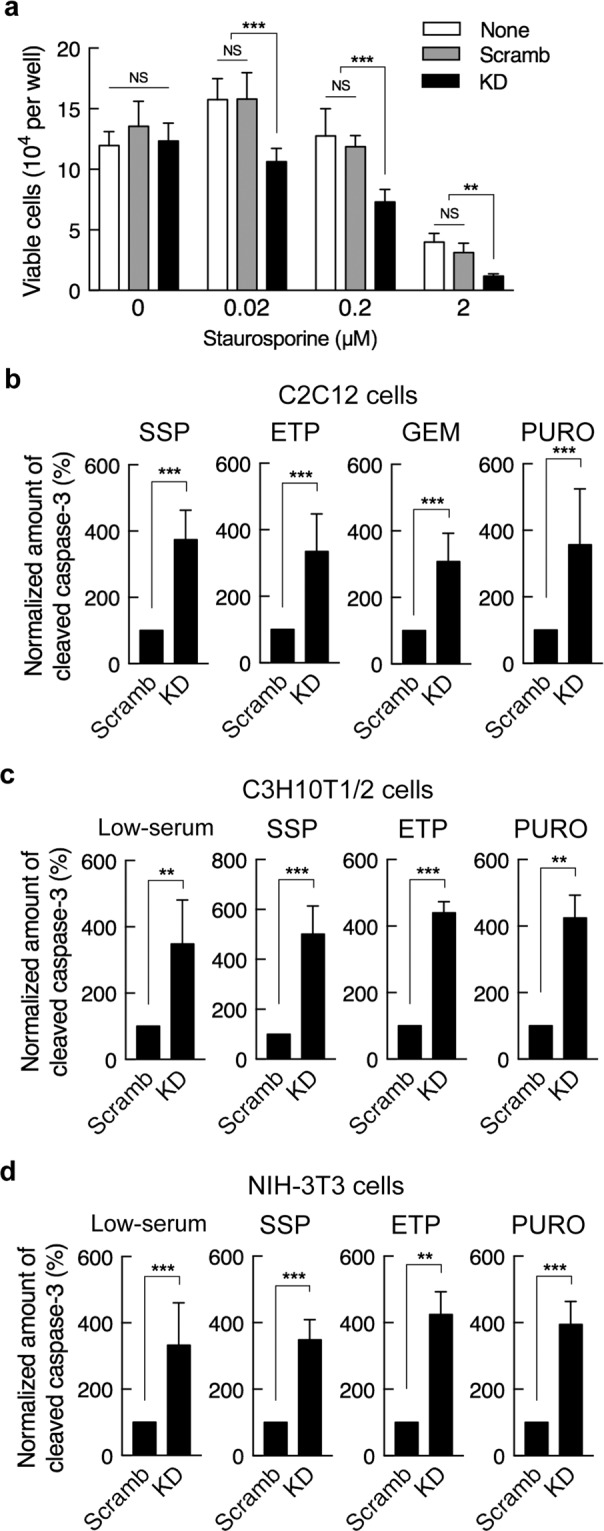
PDZRN3 depletion increases the sensitivity of cells to various apoptotic stimuli. (**a**) Viability of C2C12 myoblasts infected or not (None) with adenoviruses encoding an shRNA specific for mouse PDZRN3 mRNA (KD) or a scrambled version of this shRNA sequence (Scramb) and then incubated for 3 h in GM containing the indicated concentrations of staurosporine. Data are means ± s.e.m. for 10 biological replicates. **P < 0.01, ***P < 0.001, NS (two-way ANOVA). (**b**) C2C12 cells infected as in (**a**) were exposed to 0.5 μM staurosporine (SSP), 100 μM etoposide (ETP), 200 μM gemcitabine (GEM), or puromycin (PURO, 1 μg/ml) in GM for 3 h, after which cell lysates were subjected to immunoblot analysis with antibodies to cleaved caspase-3 and to α-tubulin. The abundance of cleaved caspase-3 was normalized by that of α-tubulin and expressed relative to the corresponding value for Scramb. Data are means ± s.e.m. for seven biological replicates. ***P < 0.001 (unpaired Student’s *t* test). (**c**,**d**) C3H10T1/2 cells (**c**) and NIH-3T3 cells (**d**) infected as in (**a**) were exposed either to low-serum medium for 6 h or to 0.5 μM staurosporine, 100 μM etoposide, or puromycin (1 μg/ml) in GM for 3 h. The abundance of cleaved caspase-3 was then determined as in (**b**). Data are means ± s.e.m. for four biological replicates. **P < 0.01, ***P < 0.001 (unpaired Student’s *t* test).

We previously showed that PDZRN3 is essential for differentiation from C2C12 myoblasts to myotubes^2,16^. Long-term serum deprivation of C2C12 myoblasts induces not only apoptosis but also entry into G_0_ phase of the cell cycle followed by myogenic differentiation^17–19^. To determine whether the regulation of apoptosis by PDZRN3 is related to myogenic differentiation, we examined the effects of other inducers of apoptosis—including staurosporine, etoposide, gemcitabine, and puromycin—that do not trigger myogenic differentiation. The loss of cell viability induced by staurosporine in C2C12 myoblasts was potentiated by depletion of PDZRN3 (Fig. 3a). Furthermore, each of the tested agents increased the abundance of cleaved caspase-3 to a greater extent in PDZRN3-depleted myoblasts than in control cells (Fig. 3b). Similar results were obtained with other cell types that express PDZRN3, including C3H10T1/2 mouse mesenchymal stem cells (Fig. 3c) and NIH-3T3 mouse embryonic fibroblasts (Fig. 3d), neither of which differentiate into myotubes in response to serum deprivation. Together, these results thus suggested that the regulation of apoptosis by PDZRN3 is independent of myogenic differentiation.

### Depletion of PDZRN3 results in down-regulation of cyclin A2 expression

**Figure 4:**
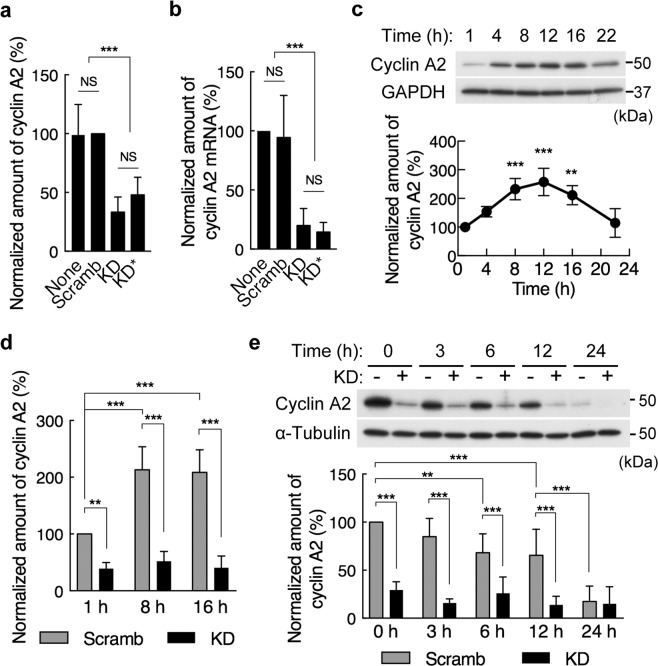
PDZRN3 depletion suppresses the expression of cyclin A2. (**a**) C2C12 myoblasts infected or not (None) with adenoviruses encoding PDZRN3 (KD or KD*) or scrambled (Scramb) shRNAs were subjected to immunoblot analysis of cyclin A2 and GAPDH (loading control). The abundance of cyclin A2 was normalized by that of GAPDH and expressed relative to the value for Scramb. Data are means ± s.e.m. for five biological replicates. ***P < 0.001, NS (one-way ANOVA). (**b**) RT and real-time PCR analysis of cyclin A2 mRNA in cells infected as in (**a**). Data are expressed relative to the value for noninfected cells and are means ± s.e.m. for five biological replicates. ***P < 0.001, NS (one-way ANOVA). (**c**) C2C12 cells synchronized by nocodazole treatment were cultured in GM for the indicated times after removal of nocodazole, after which cell lysates were subjected to immunoblot analysis with antibodies to cyclin A2 and to GAPDH. A representative blot as well as quantitative data (means ± s.e.m. for four biological replicates) for the abundance of cyclin A2 normalized by that of GAPDH and expressed relative to the value for cells at 1 h are shown. **P < 0.01, ***P < 0.001 versus cells at 1 h (one-way ANOVA). (**d**) Cells infected with adenoviruses for KD or Scramb shRNAs were analyzed as in (**c**). Data are expressed relative to the value for Scramb at 1 h after removal of nocodazole and are means ± s.e.m. for five biological replicates. **P < 0.01, ***P < 0.001 (two-way ANOVA). (**e**) Immunoblot analysis of cyclin A2 and α-tubulin (loading control) in C2C12 cells infected with adenoviruses encoding KD or Scramb shRNAs and subjected to serum deprivation for the indicated times. A representative blot as well as quantitative data (means ± s.e.m. for 10 biological replicates) for the abundance of cyclin A2 normalized by that of α-tubulin and expressed relative to the value for Scramb at time 0 are shown. **P < 0.01, ***P < 0.001 (two-way ANOVA).

We previously showed that the expression of cyclin A2 was significantly reduced at both protein and mRNA levels, whereas that of other cyclins such as cyclin E1 and cyclin D1 was unaffected, in PDZRN3-depleted C2C12 myoblasts^16^. This previous analysis was performed with confluent cells. We therefore examined the effect of PDZRN3 depletion in proliferative C2C12 cells before they achieved the confluent state. We found that depletion of PDZRN3 with either of the two shRNAs also reduced the expression of cyclin A2 at both protein and mRNA levels in the proliferating cells (Fig. 4a,b). Given that the level of cyclin A2 changes during the cell cycle, we also examined the abundance of this protein in synchronized proliferative C2C12 myoblasts. The release of control cells synchronized in M phase by exposure to nocodazole resulted in an increase in the amount of cyclin A2 that reached a peak at 12 h after removal of nocodazole (Fig. 4c). The abundance of cyclin A2 in such synchronized cells depleted of PDZRN3 was significantly smaller than that in control cells at each time point examined after removal of nocodazole (Fig. 4d). The induction of apoptosis in control C2C12 myoblasts by serum deprivation was accompanied by a decline in the abundance of cyclin A2 from an initially high level to a low level over 24 h (Fig. 4e). In PDZRN3-depleted cells, however, the amount of cyclin A2 was initially low and remained unaffected by serum deprivation (Fig. 4e).

### Depletion of PDZRN3 induces genomic instability

**Figure 5:**
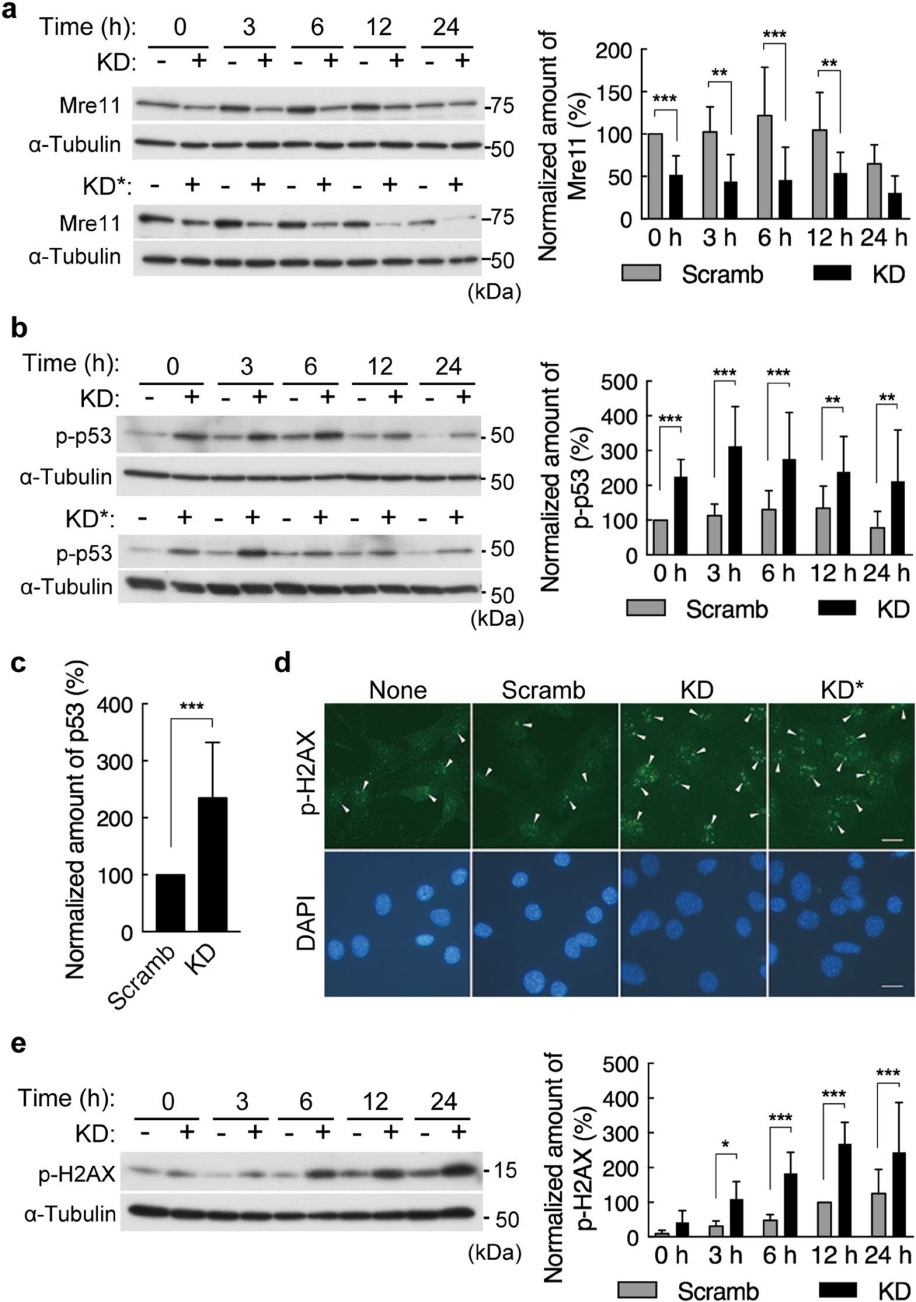
PDZRN3 depletion down-regulates Mre11, activates p53, and enhances serum deprivation-induced DNA damage. (**a**,**b**,**e**) C2C12 myoblasts infected with adenoviruses for PDZRN3 (KD or KD*) or scrambled (Scramb) shRNAs were incubated in low-serum medium for the indicated times and then subjected to immunoblot analysis with antibodies to Mre11 (**a**), to Ser^15^-phosphorylated p53 (p-p53) (**b**), to Ser^139^-phosphorylated H2AX (p-H2AX) (**e**), or to α-tubulin (loading control). Representative blots as well as quantitative data (means ± s.e.m. for 10 biological replicates) for the abundance of Mre11, p-p53, and p-H2AX normalized by that of α-tubulin and expressed relative to the value for Scramb at time 0 (**a**,**b**) or 12 h (**e**) are shown. *P < 0.05, **P < 0.01, ***P < 0.001 (two-way ANOVA). (**c**) The cells in (**b**) were also subjected to immunoblot analysis with antibodies to p53 and to α-tubulin after serum deprivation for 12 h. The abundance of total p53 protein was normalized by that of α-tubulin and is expressed relative to the value for Scramb. Data are means ± s.e.m. from nine biological replicates. ***P < 0.001 (unpaired Student’s *t* test). (**d**) C2C12 cells infected or not (None) with adenoviruses encoding PDZRN3 (KD or KD*) or scrambled (Scramb) shRNAs were subjected to immunofluorescence analysis with antibodies to p-H2AX after serum deprivation for 6 h. Nuclei were stained with DAPI. Scale bars, 25 μm. Arrowheads indicate foci of p-H2AX formed in nuclei in response to DNA damage.

Cyclin A2 was recently shown to directly activate translation of the mRNA for the DNA repair factor Mre11^20^. We observed that the abundance of Mre11 in C2C12 myoblasts fluctuated in a manner similar to that of cyclin A2 during cell cycle progression from M phase (Supplementary Fig. S1). Also like that of cyclin A2, the amount of Mre11 was significantly reduced in PDZRN3-depleted C2C12 cells (Fig. 5a). Exposure to an Mre11 inhibitor or Mre11 knockdown was also recently shown to induce the accumulation of replication stress and DNA damage in tumor cells, resulting in the activation of p53 and p53-dependent cell death^21^. Given the down-regulation of Mre11 detected in PDZRN3-depleted C2C12 cells, we examined the activation of p53 in these cells. The level of Ser^15^-phosphorylated (activated) p53 was indeed higher in PDZRN3-depleted cells than in control cells (Fig. 5b). The amount of total p53 protein was also increased in the PDZRN3-depleted cells (Fig. 5c), probably as a result of the resistance of Ser^15^phosphorylated p53 to degradation. We next evaluated DNA damage by immunofluorescence and immunoblot analyses of the Ser^139^-phosphorylated form of the histone H2A variant H2AX. Serum deprivation resulted in a time-dependent increase in H2AX phosphorylation in C2C12 myoblasts (Fig. 5d,e), consistent with previous observations^22^, and this effect was significantly augmented in PDZRN3-depleted cells compared with control cells (Fig. 5d,e). These results thus suggested that PDZRN3 might play a role in the maintenance of genomic stability by supporting the expression of Mre11 in C2C12 myoblasts.

### Restoration of cyclin A2 expression attenuates the promotion of apoptosis and the inhibition of proliferation by PDZRN3 depletion

**Figure 6:**
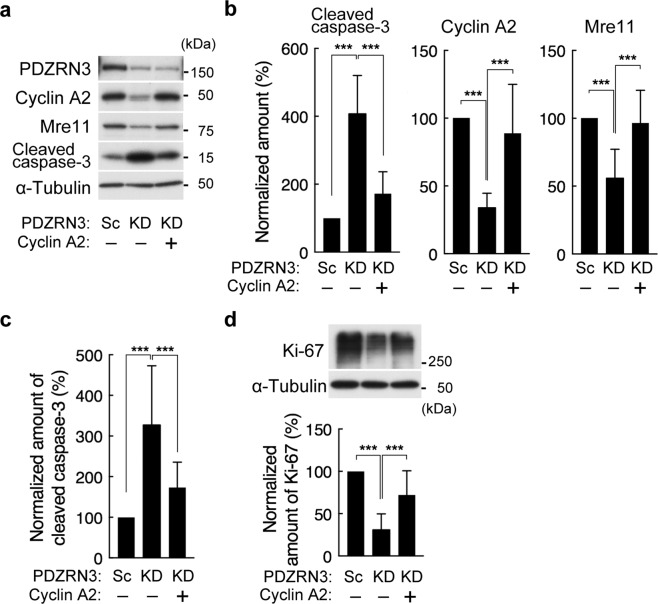
Forced expression of cyclin A2 attenuates the promotion of apoptosis and the inhibition of proliferation by PDZRN3 depletion. (**a**) C2C12 myoblasts infected with adenoviruses for PDZRN3 (KD) or scrambled (Sc) shRNAs were transfected with an expression vector for cyclin A2 (+) or with the corresponding empty vector (−) and then deprived of serum for 6 h, after which cell lysates were subjected to immunoblot analysis with antibodies to PDZRN3, to cyclin A2, to Mre11, to cleaved caspase-3, and to α-tubulin (loading control). (**b**) The abundance of cleaved caspase-3, cyclin A2, and Mre11 normalized by that of α-tubulin was determined for cells treated as in (**a**). Data are expressed relative to the value for cells infected with the scrambled shRNA and transfected with the empty vector and are means ± s.e.m. for eight biological replicates. ***P < 0.001 (one-way ANOVA). (**c**) C2C12 cells infected and transfected as in (**a**) were cultured in GM containing 0.5 μM staurosporine for 3 h and then subjected to immunoblot analysis for determination of the relative amount of cleaved caspase-3 normalized by that of α-tubulin. Data are means ± s.e.m. for 10 biological replicates. ***P < 0.001 (one-way ANOVA). (**d**) C2C12 cells infected and transfected as in (**a**) were subjected to immunoblot analysis for determination of the relative amount of Ki-67 normalized by that of α-tubulin. Data are means ± s.e.m. for 10 biological replicates. ***P < 0.001 (one-way ANOVA).

To determine whether the regulation of apoptosis by PDZRN3 is mediated by cyclin A2, we examined the effects of restoration of cyclin A2 expression in PDZRN3-depleted C2C12 myoblasts. Transfection with an expression vector for cyclin A2 restored the expression of Mre11 and attenuated the generation of cleaved caspase-3 induced by serum deprivation in PDZRN3-depleted cells (Fig. 6a,b). Similar results were obtained for cleaved caspase-3 in cells in which apoptosis was induced by exposure to staurosporine (Fig. 6c). Overexpression of cyclin A2 expression also reversed the down-regulation of Ki-67 induced by PDZRN3 depletion (Fig. 6d). These data thus suggested that depletion of PDZRN3 promotes apoptosis and inhibits proliferation in C2C12 myoblasts through down-regulation of cyclin A2 expression.

## Discussion

We have here shown that PDZRN3 regulates the expression and cell cycle-specific oscillation of cyclin A2 in C2C12 myoblasts, and that it protects these cells from apoptosis in a manner dependent on cyclin A2, likely through up-regulation of Mre11 and maintenance of genomic stability. Given that cyclin A2 is a regulator of cell cycle progression at the G_1_-S transition and mitotic entry^23–25^, PDZRN3 might also play an important role in cell proliferation in addition to apoptosis. The regulation of apoptosis by PDZRN3 was found to be independent of its role in the differentiation of myoblasts into myotubes and was also apparent in other cell types including mesenchymal stem cells (C3H10T1/2) and fibroblasts (NIH-3T3).

PDZRN3 possesses two distinct PDZ domains, whereas cyclin A2 contains a class 2 consensus binding sequence for the PDZ domain in its COOH-terminal region. The two proteins might thus interact with each other through at least one of the PDZ domains of PDZRN3. However, it is not likely that PDZRN3 regulates the abundance of cyclin A2 via direct interaction between the two proteins, given that no such physical interaction was detected by immunoprecipitation (Supplementary Fig. S2) and that PDZRN3 depletion was shown in the present study to reduce the amount of cyclin A2 mRNA in C2C12 myoblasts. It is possible that the regulation of cyclin A2 expression by PDZRN3 is mediated by another molecule, the identity of which remains to be determined.

Mre11 plays an important role in the repair of DNA damage and consequent maintenance of genomic stability^26^. Depletion of PDZRN3 resulted in down-regulation of both cyclin A2 and Mre11 in C2C12 myoblasts. Given that forced expression of cyclin A2 in PDZRN3-depleted cells restored the expression of Mre11, the loss of cyclin A2 might be responsible for the down-regulation of Mre11. Indeed, the expression of Mre11 has been shown to be controlled by cyclin A2^20^. The down-regulation of Mre11 was associated with an increase in the extent of DNA damage and apoptosis induced by serum deprivation. In addition, phosphorylation of Akt at Ser^473^ was inhibited in PDZRN3-depleted cells. The complex of cyclin-dependent kinase 2 and cyclin A2 has been shown to directly interact with Akt and phosphorylate it at Ser^477^ and Thr^479^, with such phosphorylation promoting that at Ser^473^ by mTORC2^27^. The enhancement of apoptosis in PDZRN3-depleted cells might thus result in part from a reduction in the extent of cyclin A2-dependent Akt phosphorylation in addition to the down-regulation of Mre11.

In the present study, we have shown that depletion of PDZRN3 promotes apoptosis and DNA damage. Although apoptosis-related signaling and DNA damage are required for differentiation from myoblasts to myotubes^22,28–31^, such differentiation is inhibited by depletion of PDZRN3^2,16^. This apparent inconsistency might be explained in part by our previous observation that the inhibition of such differentiation by PDZRN3 depletion is mediated by the up-regulation of Id2^16^. DNA damage before the induction of myoblasts to myotubes differentiation has also been shown to inhibit the differentiation process^32,33^.

The effects of PDZRN3 depletion on apoptosis (Fig. 3c,d) and the expression of cyclin A2 and Mre11 (data not shown) apparent in C2C12 myoblasts were also detected in other cell types including C3H10T1/2 and NIH-3T3 cells. The regulation of apoptosis and proliferation by PDZRN3 might thus be common among cells that express this protein. It will be of interest to examine whether signaling from PDZRN3 is altered in tumor cells. Various mutations of the PDZRN3 gene have been identified in several tumor types^10–15^, although it remains unknown how these mutations affect tumor development and progression. Further studies are warranted to determine the role of PDZRN3 in tumor cells.

## Methods

### Materials

Staurosporine was obtained from LC Laboratories (Woburn, MA), etoposide was from Cayman Chemical (Ann Arbor, MI), gemcitabine was from Toronto Research Chemicals (Toronto, ON, Canada), and nocodazole, puromycin and thymidine were from Sigma-Aldrich (St. Louis, MO). BrdU was obtained from BD Pharmingen (San Diego, CA). Polyclonal antibodies to PDZRN3 were generated and purified as described previously^16^. Rabbit polyclonal antibodies to cyclin A2 (GTX103042) and to Mre11 (GTX118741) were obtained from GeneTex (Irvine, CA), those to cytochrome c (#21680) were from Signalway Antibody (College Park, MD), those to glyceraldehyde-3-phosphate dehydrogenase (GAPDH, 2275-PC) were from Trevigen (Gaithersburg, MD), and those to cleaved caspase-3 (#9661), to Ser^15^-phosphorylated p53 (#9284), to Ser^473^-phosphorylated Akt (#9271), and to Akt (#9272) were from Cell Signaling Technology (Danvers, MA). Mouse monoclonal antibodies to α-tubulin (T-9026), to p53 (#2524), to Ser^139^-phosphorylated H2AX (GTX628789), and to BrdU (66241-1-Ig) were from Sigma-Aldrich, Cell Signaling Technology, GeneTex, and Proteintech group (Chicago, IL), respectively, and rabbit monoclonal antibodies to Ki-67 (RM-9106-R7) were from Thermo Fisher Scientific (Waltham, MA). Phosphatase and protease inhibitor cocktails were obtained from Roche (Branchburg, NJ).

### Cell culture

The mouse myoblast cell line C2C12 (ATCC-CRL-1772), mouse mesenchymal stem cell line C3H10T1/2 (ATCC-CCL-226), and mouse embryonic fibroblast cell line NIH-3T3 (ATCC-CRL-1658) were obtained from American Type Culture Collection (Manassas, VA) and were maintained at 37 °C under 5% CO_2_ in GM consisting of Dulbecco’s modified Eagle’s medium supplemented with 10% fetal bovine serum, penicillin (100 U/ml), and streptomycin (100 μg/ml). Cells were subjected to serum deprivation by culture in medium containing 1% fetal bovine serum.

### RNAi

Recombinant adenoviruses encoding shRNAs specific for mouse PDZRN3 mRNA (KD and KD*) or an shRNA corresponding to a scrambled version of KD (Scramb) were constructed as described previously^16^. Unless indicated otherwise, C2C12 cells were seeded at a density of 2 × 10^4^ cells per well in a 48-well plate, cultured in GM for 3 h, and infected with adenoviruses at a multiplicity of infection of 100 plaque-forming units per cell for 24 h, after which the medium was replaced with fresh GM and the cells were cultured for 24 h to ~90% confluence before analysis. C3H10T1/2 and NIH-3T3 cells were seeded, infected, and cultured in the same manner as C2C12 cells with the exception that the multiplicity of infection was 20 and 30 plaque-forming units per cell, respectively.

### Cell viability assay

C2C12 cells infected with adenoviruses for 48 h were reseeded at a lower density (0.5 × 10^4^ cells per well in a 48-well plate), cultured for 48 h, collected by exposure to trypsin, and washed with phosphate-buffered saline (PBS). Nonviable cells were distinguished from viable cells by staining with trypan blue dye (Sigma-Aldrich). For examination of the effect of staurosporine on C2C12 cell viability, the infected cells were reseeded at 2.5 × 10^4^ cells per well in a 48-well plate, cultured for 12 h, and then exposed to staurosporine for 3 h before determination of the proportion of viable cells.

### Cell adhesion assay

C2C12 cells infected with adenoviruses for 48 h were reseeded at 2.5 × 10^4^ cells per well in a 48-well plate that had been coated with 0.2% gelatin in PBS and were cultured for 1 h, after which the proportion of cells attached to the plate was determined.

### BrdU incorporation assay

C2C12 cells infected with adenoviruses for 48 h (2.5 × 10^4^ cells) were re-seeded on 15-mm cover glasses that had been coated with 0.2% gelatin in PBS and placed in the wells of a four-well plate. After cultured for 24 h, the medium was replaced with GM containing 10 µM BrdU and the cells were cultured for 12 h before fixation with 4% paraformaldehyde for 20 min, permeabilization with 0.5% Triton X-100 in PBS for 5 min, and treatment with 2 M HCl for 60 min at room temperature. The cells were then exposed to 1% bovine serum albumin for 30 min before incubation for 60 min at room temperature first with antibodies to BrdU (1:500 dilution) and then with Alexa Fluor 488-conjugated goat antibodies to mouse immunoglobulin G (Molecular Probes, Eugene, OR). The cover glasses were mounted with the use of Vectashield mounting medium containing 4′,6-diamidino-2-phenylindole (DAPI; Vector Laboratories, Burlingame, CA) and examined with a fluorescence microscope (Axiovert S1000; Carl Zeiss, Jena, Germany). The proportion of cells positive for BrdU among all DAPI-positive cells was determined with the use of Image J software (NIH, Bethesda, MD).

### Apoptosis assay based on annexin V staining

C2C12 cells infected with adenoviruses for 48 h (2.5 × 10^4^ cells) were re-seeded on 15-mm cover glasses that had been coated with 0.2% gelatin in PBS and placed in the wells of a four-well plate. After cultured for 24 h, the cells were deprived of serum for 6 h before assay of apoptosis with the use of an Annexin V-FITC apoptosis detection kit (Nacalai tesque, Kyoto, Japan). The stained cells were washed with annexin binding buffer and fixed with 4% paraformaldehyde for 20 min. The cover glasses were mounted with the use of Vectashield mounting medium containing DAPI (Vector Laboratories) The percentage of early apoptotic (annexin V-positive, propidium iodide-negative) cells was determined with a fluorescence microscope (Axiovert S1000; Carl Zeiss, Jena).

### RT and real-time PCR analysis

Total RNA was isolated from C2C12 cells with the use of an SV Total RNA Isolation System (Promega, Madison WI) and was subjected to reverse transcription (RT) with the use of a ReverTra Ace qPCR RT Kit (Toyobo, Osaka, Japan). The resulting cDNA was subjected to real-time polymerase chain reaction (PCR) analysis with a FastStart Universal SYBR Green Master Kit (Roche) and an Applied Biosystems StepOne Plus Real-Time PCR System (Life Technologies, Carlsbad, CA). The relative abundance of cyclin A2 mRNA was estimated with the ΔΔCt method and with GAPDH mRNA as an internal control. The sequences of the PCR primers (sense and antisense, respectively) were 5′-CTTCTTCCTTTTCCCTTGGC-3′ and 5′-TTTCAGAGTCCCAGTGACCC-3′ for mouse cyclin A2, and 5′-CTCCCACTCTTCCACCTTCG-3′ and 5′-CATACCAGGAAATGAGCTTGACAA-3′ for mouse GAPDH.

### Cell cycle synchronization

Cell synchronization at G_2_-M was performed by a modified version of the thymidine-nocodazole block protocol^34^. C2C12 myoblasts were seeded at a density of 1.5 × 10^4^ cells per well in a 48-well plate, cultured in GM for 3 h, and infected with adenoviruses at a multiplicity of infection of 100 plaque-forming units per cell for 9 h. They were then treated consecutively with 2 mM thymidine for 21 h and with 1 µM of nocodazole for 12 h for synchronization. The cells were collected for analysis at various times after removal of nocodazole.

### Overexpression of cyclin A2

C2C12 cells (2.5 × 10^4^ cells per well of a 48-well plate) were infected with adenoviruses for 12 h and then transfected with a pcDNA3.1(+) vector (300 ng) encoding mouse cyclin A2 (or with the empty vector as a control) by the one-step method with the use of a ScreenFect A *plus* transfection reagent kit (Wako, Osaka, Japan).

### Immunoblot analysis

C2C12 myoblasts were washed twice with Tris-buffered saline and then lysed in a solution containing 150 mM NaCl, 20 mM Tris-HCl (pH 7.4), 20 mM Na_3_VO_4_, 20 mM NaF, 0.3% Triton X-100, and phosphatase and protease inhibitor cocktails. The lysates were subjected to immunoblot analysis as described previously^16^.

### Analysis of cytosolic cytochrome c

C2C12 myoblasts were washed twice with a solution containing 150 mM NaCl, 2 mM CaCl_2_, and 20 mM Tris-HCl (pH 7.5), scraped into a solution containing 150 mM NaCl, 2 mM dithiothreitol, 20 mM Tris-HCl (pH 7.5), and a protease inhibitor cocktail, and homogenized with the use of a Polytron disrupter (Kinematica AG, Lucerne, Switzerland). The homogenate was centrifuged at 500 × *g* for 10 min at 4 °C, and the resulting supernatant was centrifuged at 100,000 × *g* for 30 min at 4 °C to yield a cytosolic fraction (supernatant). Equal amounts of whole-cell and cytosolic protein were subjected to immunoblot analysis with antibodies to cytochrome c.

### Immunofluorescence analysis

Cells grown on 15-mm cover glasses coated with 0.2% gelatin were fixed with 4% paraformaldehyde for 20 min at room temperature, permeabilized for 5 min with PBS containing 0.5% Triton X-100, and exposed for 30 min to 3% bovine serum albumin before incubation for 60 min at room temperature with antibodies to Ki-67 (1:100 dilution) or to phospho-H2AX (1:100 dilution). Immune complexes were detected by incubation of the cells for 30 min at room temperature with Alexa Fluor 488-conjugated goat antibodies to rabbit or mouse immunoglobulin G (Molecular Probes, Eugene, OR). The cover glasses were mounted with the use of Vectashield mounting medium containing DAPI (Vector Laboratories) and examined with a fluorescence microscope (Axiovert S1000, Carl Zeiss). The proportion of cells positive for Ki-67 was determined with the use of Image J software (NIH).

### Statistical analysis

Data are presented as means ± s.e.m. and were compared between two groups with the unpaired Student’s t test and among three or more groups by one-way or two-way analysis of variance (ANOVA) followed by the Tukey-Kramer post hoc test. Statistical analysis was performed with the use of Prism 7 version 7.0b software (GraphPad Software, La Jolla, CA). A P value of <0.05 was considered statistically significant.

## Supplementary information

Supplementary Information.

## Data Availability

All data generated or analyzed during this study are included in this published article and its Supplementary Information file.
