# Supplementary material for: PDZRN3 protects against apoptosis in myoblasts by maintaining cyclin A2 expression
Source: Sci Rep. 2020 Jan 24;10:1140. doi: 10.1038/s41598-020-58116-1 (PMC6981127; doi:10.1038/s41598-020-58116-1)
Supplement: Supplementary file 1 — Supplementary Information. [file 41598_2020_58116_MOESM1_ESM.pdf]

Supplementary Information

PDZRN3 protects against apoptosis in myoblasts by maintaining cyclin A2 expression

Takeshi Honda[1] & Makoto Inui[1,2]*

[1]Department of Pharmacology, Yamaguchi University Graduate School of Medicine, Ube, Yamaguchi 755-8505, Japan.
[2]Present address: YIC Rehabilitation College, 4-11-1 Nishiube-Minami, Ube, Yamaguchi 759-0208, Japan.
*Correspondence and requests for materials should be addressed to M.I. (email: minui@yamaguchi-u.ac.jp)

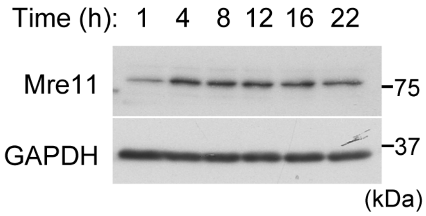

Supplementary Figure S1. Changes in Mre11 abundance during the cell cycle. C2C12 cells synchronized by nocodazole treatment were cultured in GM for the indicated times after removal of nocodazole, after which cell lysates were subjected to immunoblot analysis with antibodies to Mre11 and to GAPDH (loading control). A representative blot from three biologically independent experiments is shown. The abundance of Mre11 fluctuated in a manner similar to that for cyclin A2 during cell cycle progression from M phase (Fig. 4c).

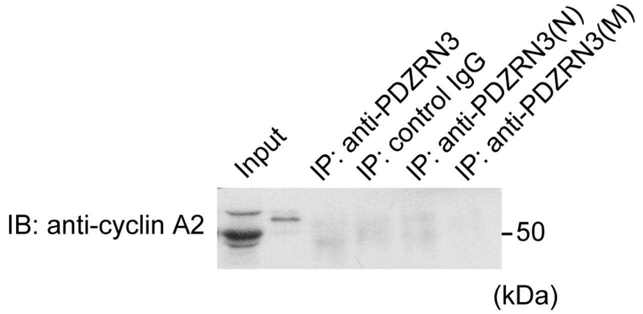

Supplementary Figure S2. Cyclin A2 was not co-immunoprecipitated with PDZRN3. A lysate of C2C12 cells was subjected to immunoprecipitation (IP) with mouse antibodies to PDZRN3, rabbit anti-PDZRN3 polyclonal antibodies (N and M) or control immunoglobulin G (IgG), and the resulting precipitates as well as the original cell lysate (Input) were subjected to immunoblot (IB) analysis with antibodies to cyclin A2. N and M recognize the N-terminal and middle region of PDZRN3, respectively.

Fig. 1a

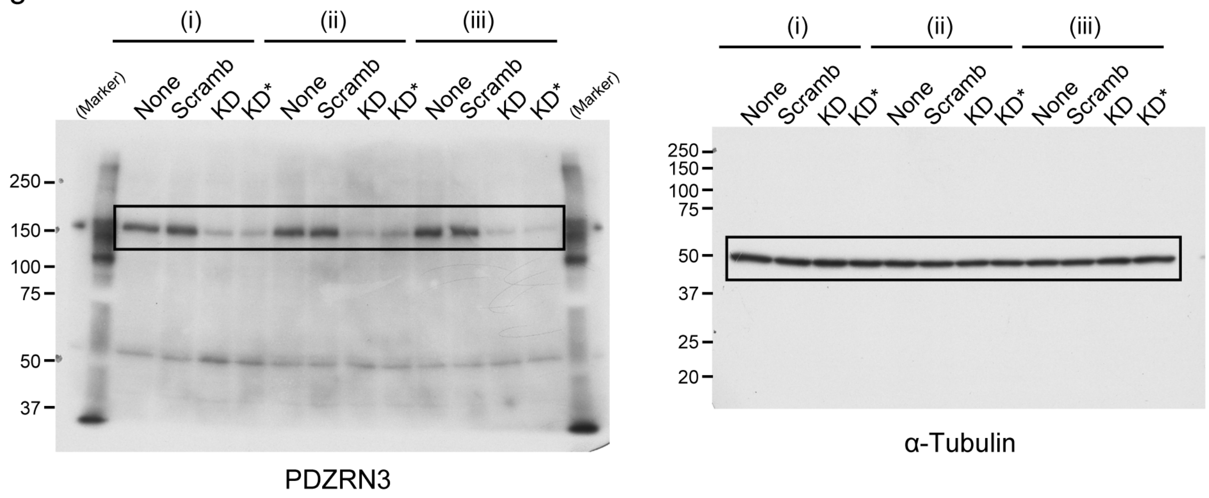

Fig. 1h

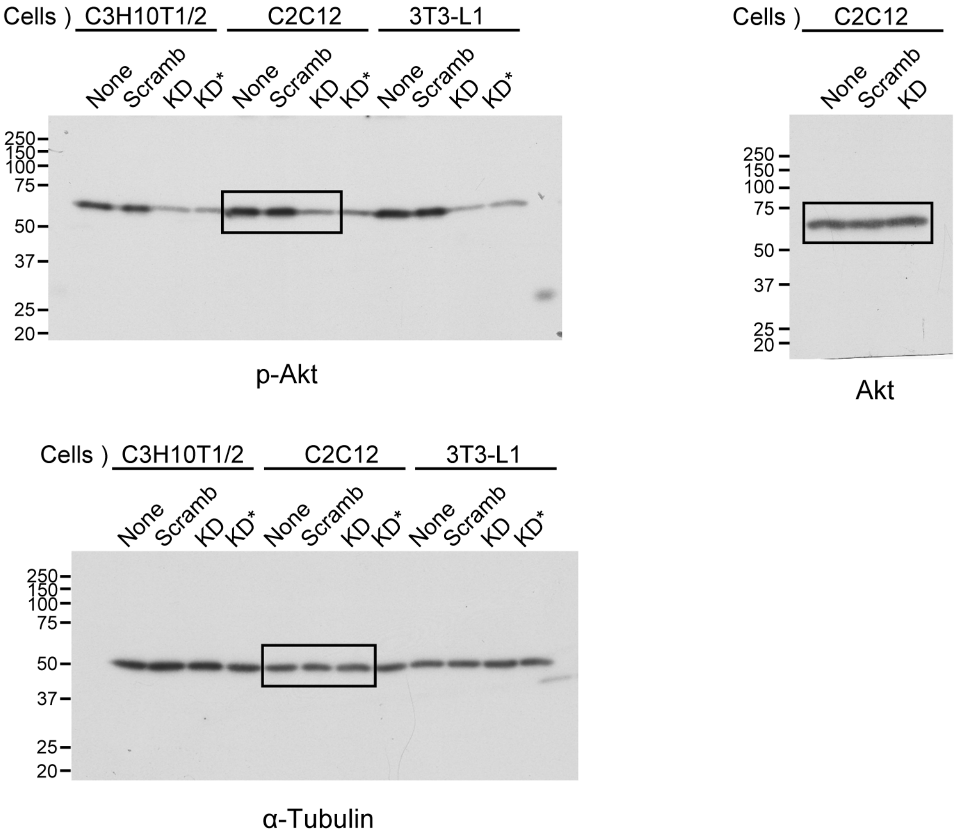

Supplementary Figure S3. Original blots regarding to data shown in Figure 1.

Fig. 2a

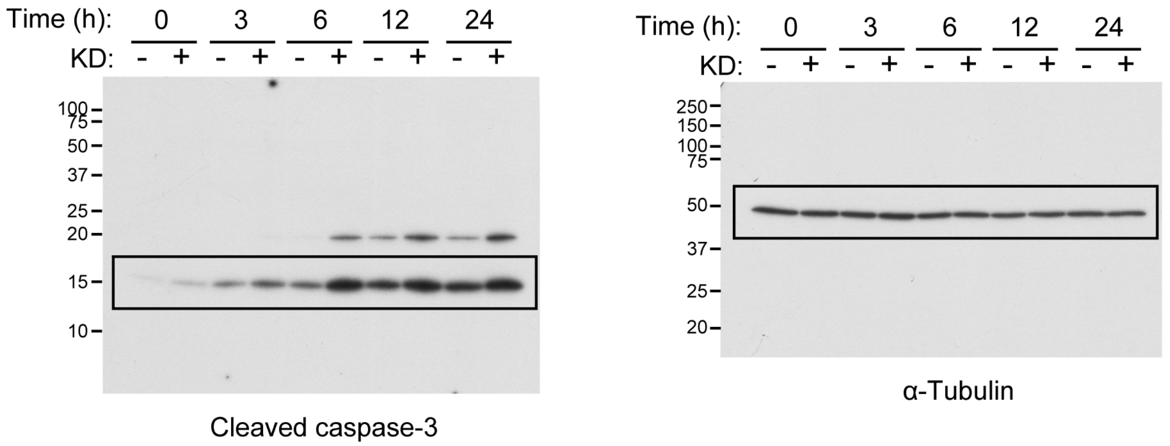

Fig. 2d

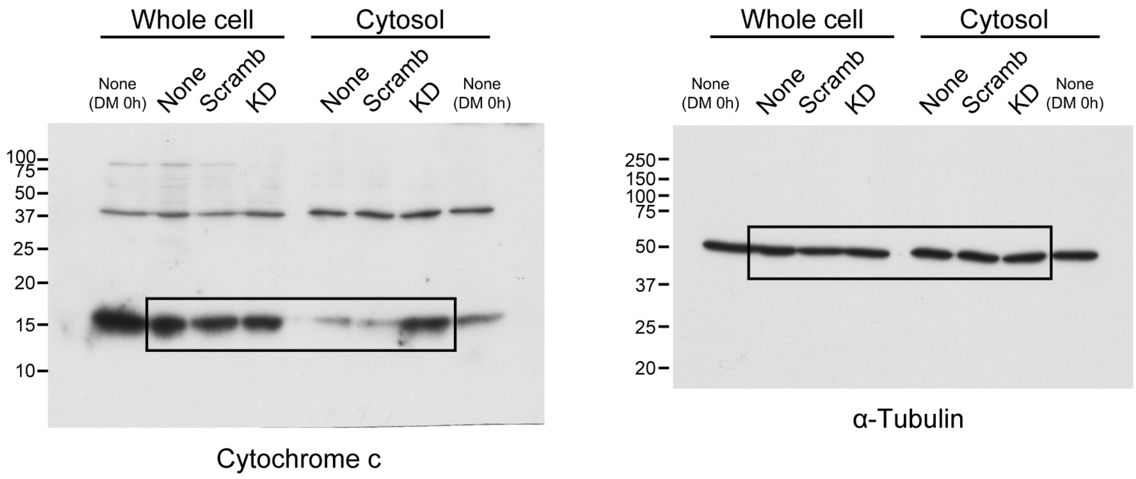

Supplementary Figure S4. Original blots regarding to data shown in Figure 2.

Fig. 4c

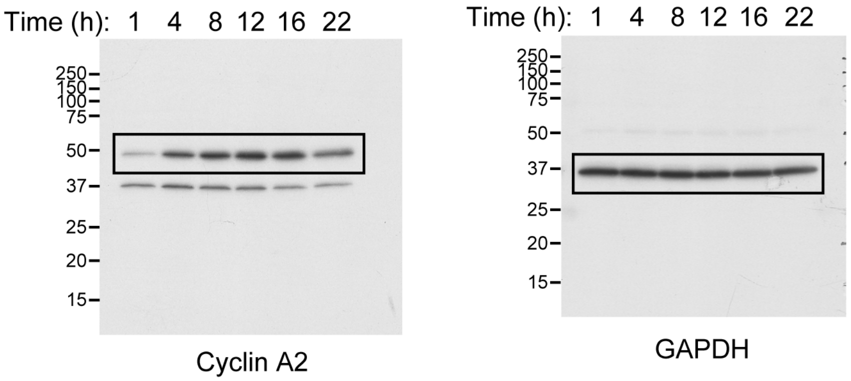

Fig. 4e

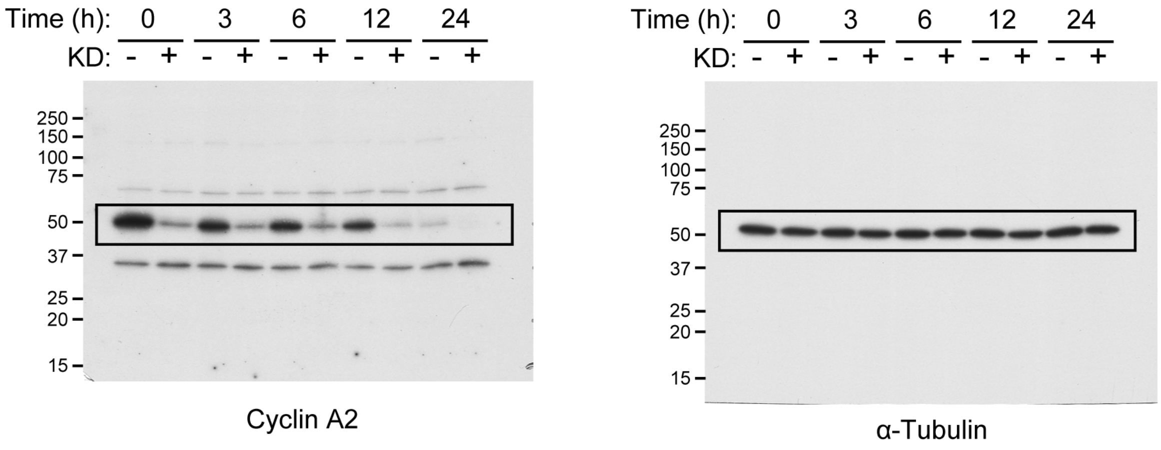

Supplementary Figure S5. Original blots regarding to data shown in Figure 4.

Fig. 5a

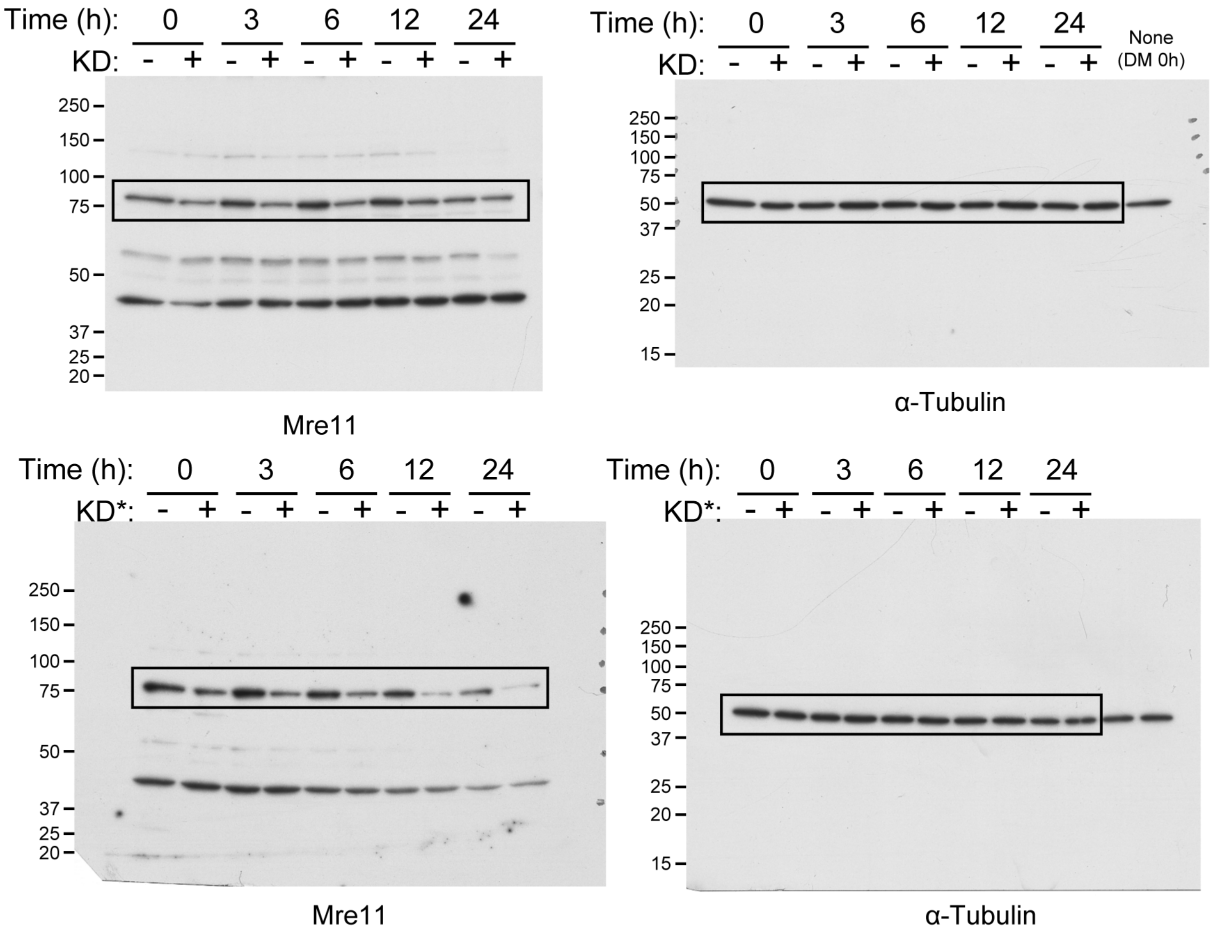

Supplementary Figure S6. Original blots regarding to data shown in Figure 5a.

Fig. 5b

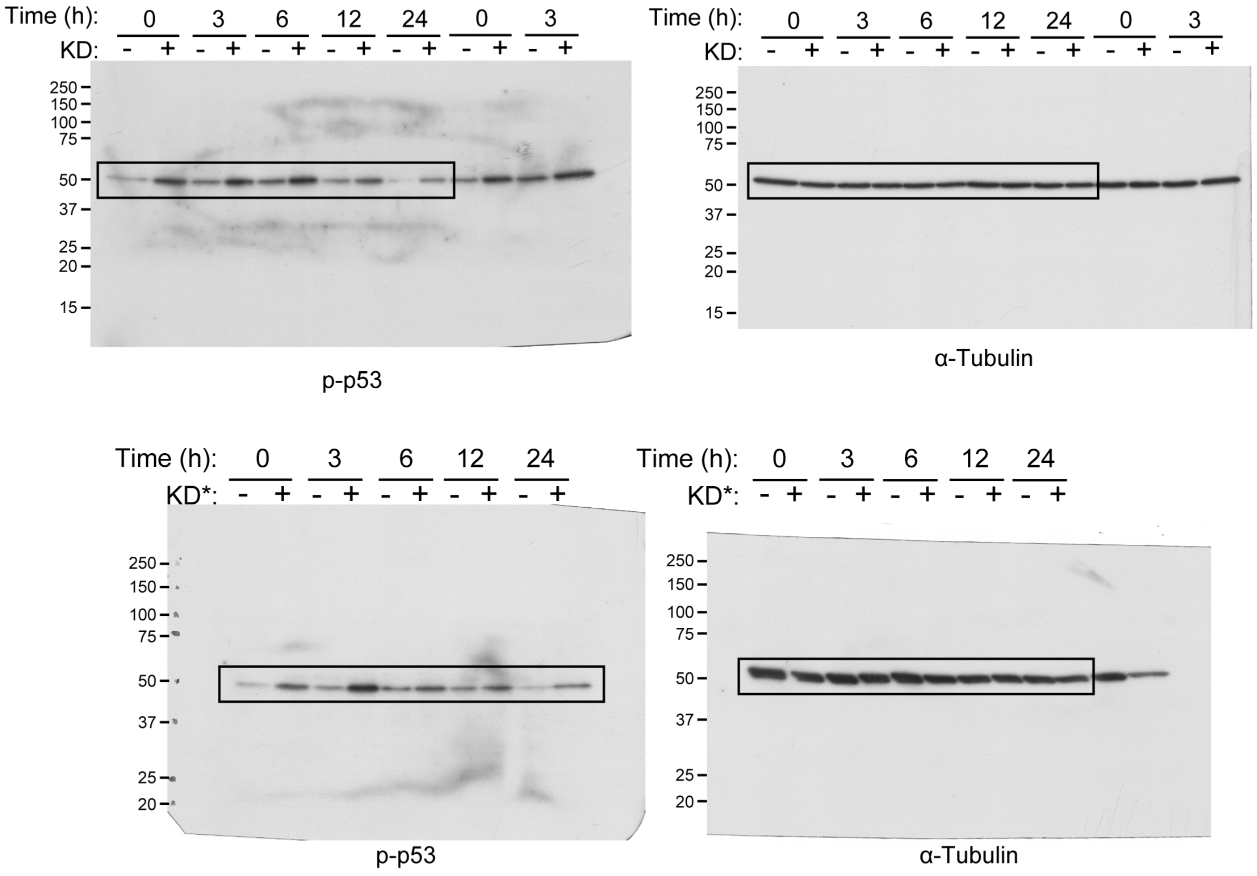

Supplementary Figure S7. Original blots regarding to data shown in Figure 5b.

Fig. 5e

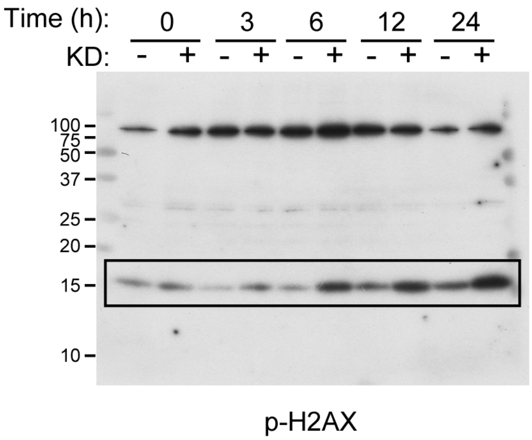

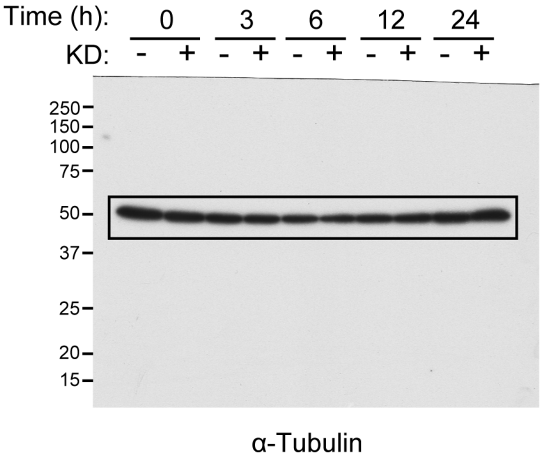

Supplementary Figure S8. Original blots regarding to data shown in Figure 5e.

Fig. 6a

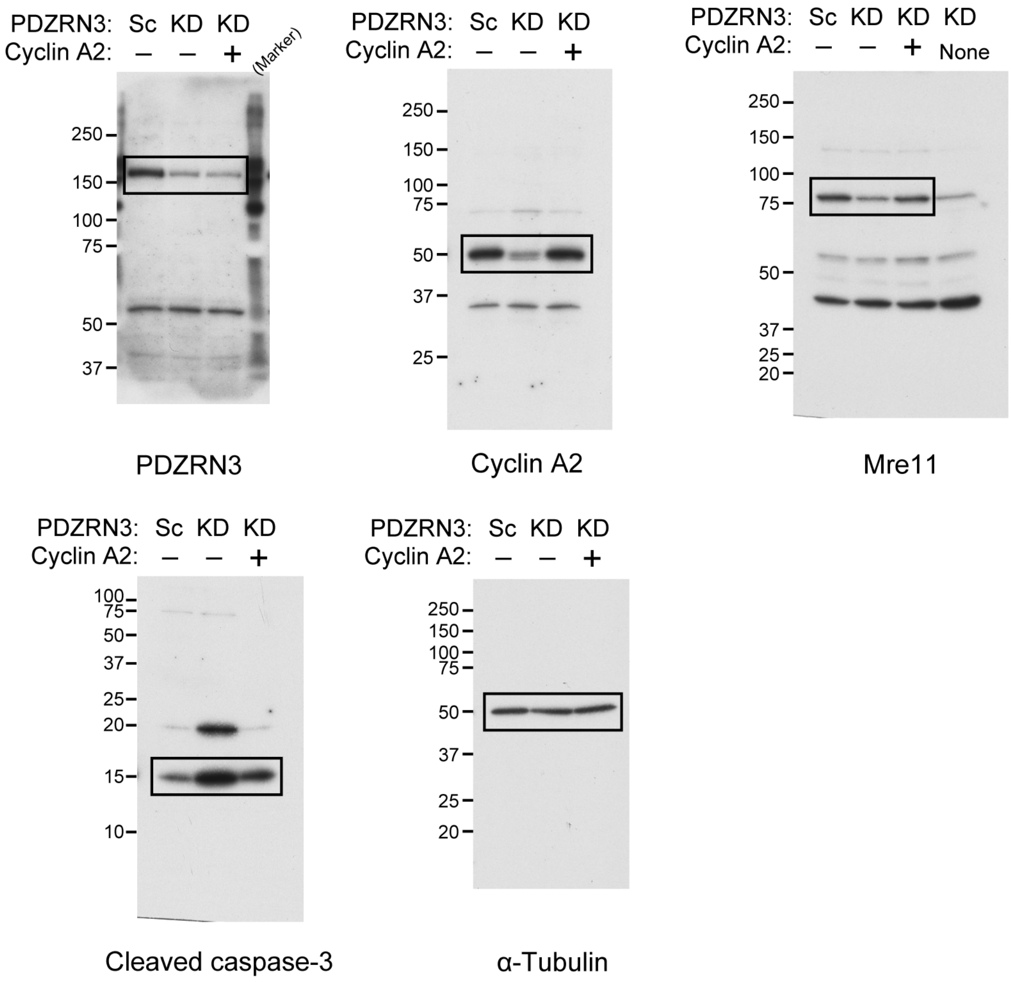

Fig. 6d

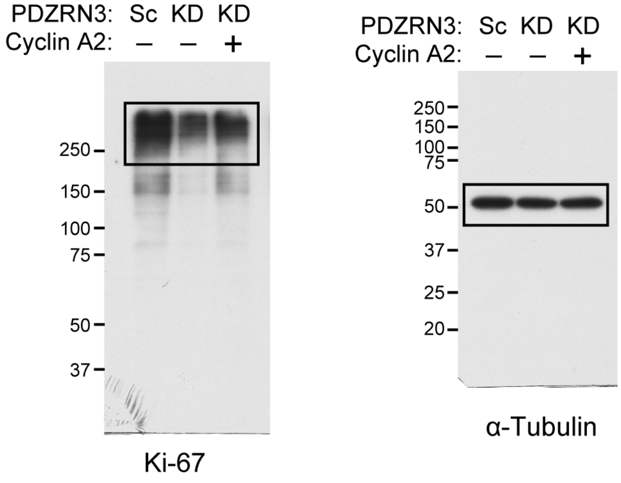

Supplementary Figure S9. Original blots regarding to data shown in Figure 6.
